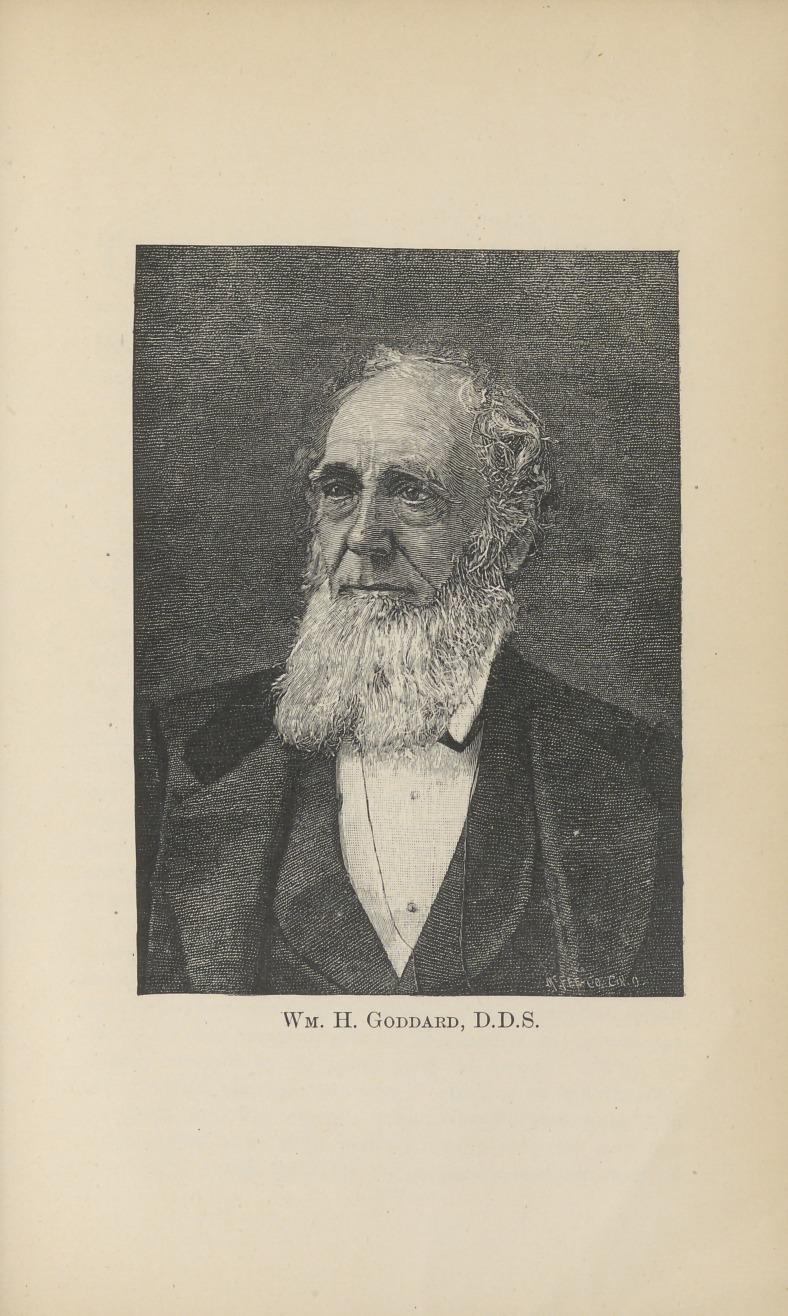# Wm. H. Goddard, D.D.S., Louisville, Kentucky

**Published:** 1883-07

**Authors:** 


### THE DENTAL REGISTER.

VOL. XXXVII.] JULY, 1883. [No. 7.

### BIOGRAPHICAL.

### Wm. H. Goddard, D.D.S., Louisville, Ky.

Wm. H. Goddard, D.D.S., of Louisville, Kentucky, was the twelfth and youngest child of Dr. Thatcher Goddard, formerly a physician, but later in life a merchant in Boston, Mass.

He was born in that city, on the 25th day of June, 1808, and reared in affluence; nothing which money and loving and devoted parents could devise for his good were denied him. Every advantage for obtaining a thorough classical education was given him, but, like too many young men, he did not appreciate his opportunities, but suffered them to pass unimproved, preferring play to study. At the proper age he attended the Latin school in that city, then under the charge of Benjamin Gould, Esq., and became somewhat a proficient in the Latin and Greek languages. After leaving that school he attended the Military school, under the charge of Capt. Alden Partridge, (who formerly had charge of the West Point Military School, of New York), located in the town of Norwich, Vermont. When he entered that school he was the youngest of 180 students; only fifteen years of age; and the shortest in stature; standing when in line on the extreme left, and was the first boy from the city of Boston. On his return from school, during his first vacation, with his regementals on, he was continually accosted by the ladies upon the street: "Why, my young soldier, where are you from?" Having an impediment in his speech, he would respond: "Nor, Nor, Norwich cadet." Invitations from many of the wealthy families, who had sons, were sent to him to visit them, for the purpose of learning about the school, and when he returned, forty-five new cadets accompanied him.

The school was a good one for boys who were studious, but the reverse for idlers, although strict military discipline was continually kept up. Many severe fights between the students at Dartmouth College, at Hanover, New Hampshire, just across the river, and the cadets took place, the latter always the victors.

Having remained two years, he returned to Boston, and, desiring to be a farmer, his father took him to Dunstable, New Hampshire, to a Mr. Ingalls, who farmed in the summer and was a blacksmith in the winter. He made only axes, hatchets, hammers and hoes. There is no doubt that his father desired that his son should experience the hardships of a farmer upon a New England soil, which is one-half stone, and only little can be raised with constant care and attention. But the son entered into it with zest, and blowed the bellows and swung the sledge with vigor all winter, and assisted in making the articles, which were Mr. Ingall's specialty. During the winter he had learned, and did make with his own hands, an axe, hammer, hatchet and hoe, for his own use. During the spring and summer, he went into the field with the same ardor, accompanied by his boss to till the soil. There he followed the plough, swung the scythe, used the sickle and gathered the harvest, but soon became dissatisfied with such a life and returned home. The farmer was very strict, allowing only so much at meals, and at such an hour he must retire and be up at dawn of day.

After his return he entered as clerk in the store of a commerce merchant, who owned many ships and brigs, trading with all parts of the world.

At the age of nineteen, the spirit of adventure and doing for himself possessed him and he left Boston for New York to seek his fortune, against the wish of his parents. In 1828 he entered the office of a dentist in that city to learn his profession. No colleges or other facilities to learn dentistry at that time. Every office and laboratory was sealed to prevent intrusion, no instruction to be obtained from any one save the one he was with, and what he could teach was very little. The student in those days was obliged to plod along as best he could, gleaning a little here and there, but mostly dependent upon his own genius. Research was out of the question, no books, periodicals, or teachers. Dr. G. fortunately possessed talent and genius, and was able to overcome in many ways, obstacles which would present themselves, which his preceptor could not unfold.

He has related many times to his professional brethren the feelings and pleasure he experienced in extracting his first tooth. His preceptor was absent for a few days; during the time, a gentleman called suffering with tooth ache.

"Is the doctor in?"

No, sir, was the response.

"I want a tooth extracted"

Take the chair, sir, I can extract it as well as the doctor. He took it, and I adjusted my instruments, looked into his mouth and found a wisdom tooth badly decayed. The instruments in those days were very different from those now in use. The one used was constructed with two parallel rods about six inches long, with handles and beaks extending downward at right angles; opening the handles opened the beaks—a piece of wood for a fulcrum was placed behind the beaks when a tooth was to be extracted, if a tooth was behind it; if not, the wood was placed in front, this being a wisdom tooth in the upper jaw, the fulcrum was placed in front. I began to be scared, and found my hand shook considerably—went to the window, looked out, found my patient was a doctor, he was a student once, and there must be a first time I thought, and he is just the man to commence upon—got hold, stood up behind him, took hold carefully and pulled up, pop came the tooth, and I grew two feet in five minutes. The patient exclaimed: "admirably done, the easiest tooth I ever had pulled." The ice was broken and fear vanished.

After remaining one year, and paying his tuition fee, he was told he was competent to practice. He opened an office up town and waited for his victims. His first patient was a gentleman for whom he had done some work while with his preceptor; he was very particular with his teeth, and a filling had come out, and he wished it replaced. Showing the filling, he asked: "What makes this gold look so dark?" After examining it carefully, the doctor responded, this is tin foil. "What, said the patient, I paid the doctor for gold, and would have nothing else in my teeth." Yes, tin foil enveloped in gold, is what doctor ——— generally used. "Are my teeth filled with that altogether?" I presume so, as it was his general practice. "I wish you to remove every filling I have in my mouth, and replace with gold," which was done. That job was completed, and the only one the doctor had, except the extraction of a few teeth, for six months.

Finding his practice as a dentist not very renumerative, he must find something else to do for bread and butter. One evening he strolled into a small drug store, owned by an uncle of a young lady he was visiting, and afterwards his wife, a Miss Graham, of New York. He proposed purchasing his stock, etc., asking the gentleman, "what will you take for this store as it is?" The man was surprised, for he had no intention of selling, and responded, "it is not for sale," but the doctor insisted, "name your price and terms," one thousand dollars," thinking to scare the doctor off, three, six, nine, and twelve months." The store is mine responded the doctor, make out a bill of sale, write the notes and I will sign them. Turning to the clerk he said, how much are you paid? Will you remain with me for the same? "Yes." You are my clerk. Make out a list of articles you need in the store to-morrow, and I will get them. On the next day the articles were purchased upon time and a good retail stock was on hand. Suffice it to say, by strict attention to business, (his wife being cashier,) the whole was paid for when the notes fell due. Keeping the store a few years, his pratice gradually increased, and his time was required in the office. He sold out, and, with a few thousand dollars migrated to Illinois, by canal boat to Buffalo, steamer to Detroit, and wagon to Chicago, said city containing only a few hundred inhabitants; went from there to the interior, Ottawa, a very small place of a few log houses, boarded his family in one of them, which was kept by a Mrs. Pembroke, consisting of only one room, with three other families, each one occupying a corner, and the lady who owned it took lodgers besides. Making his family comfortable, the Dr. commenced prospecting, and finally squatted upon a one-half section of land, about twelve miles from Ottawa, and camped out from November to the middle of January, 1834, while building a log house, his nearest neighbor being six miles. There being no mills to make lumber, puncheon floors and clapboards rived out of logs were necessary, with quilts and blankets for doors. His family soon moved in and began a life upon the prairies, the place he called Brookfield.

He purchased oxen, cows, hogs, etc., and for two years lived upon pork and cornmeal with no vegetables.

The great land sale held at Chicago he attended, bought his land at $1.25 per acre, and was present when the school section in Chicago was sold for $28.00 per acre. He has often said if he had only bought ten acres, and fallen asleep like Rip Van Winkel, when he awoke he would have been a rich man.

In farming the doctor was unfortunate, the laborers digging the canal from Chicago to Peru stole his hogs, his horses and oxen died, and three of his family, wife, daughter four years old, and a son ten months; all within one year, and had only one child left. He became disheartened, sold what little he had, paid his debts, and with his wife's mother, sister and son, left for New York. Arriving at Chicago, he found he had not money enough to pay the passage of all. He put all but himself on a steamer for Buffalo, giving them all the money he had but fifty cents, and said, Good Bye. Having his instruments, some teeth, and a little gold and tin foil, he commenced practice in Chicago; was obliged to do some things that would now hardly be regarded professional to get along, but succeeded in making a few hundred dollars, when he left for New York. After visiting his friends in New York, he came to Louisville to see his brother, a prominent teacher in that city, and concluded to locate there in 1837. He in a short time succeeded in establishing himself in a large and lucrative practice which he fully sustained for about twenty years.

In 1856 he relinquished his profession and embarked with a dear friend in manufacturing agricultural implements, and prosecuted that business with success until the late war of 1860, when their trade (which was principally with the South) ceased. A large debt due the firm was lost, their bills (which had been discounted by the banks) were returned to them, and they were obliged to pay both sides of the ledger. This misfortune came very near driving them to the wall, but through the leniency of the banks, and their other creditors, they were able to stem the torrent, and finally paid every cent with interest. During the war their manufactory was used by the Government as a hospital for sick and wounded soldiers. and the doctor and his wife devoted all their time among the hospitals, in endeavoring to ameliorate the condition of the sufferers within them.

He was instrumental in establishing a subsistence committee at the Nashville Depot, where several thousand soldiers were fed, as they passed through the city on their way south. The doctor was unreservedly for the Union, as was also his family. He was president of the Dickens club, a strong amateur company, consisting of the elite in the city, and thousands of dollars were raised by their entertainments for the families of the volunteer soldiers, sanitary commission, and other charities.

During the war he was deputy collector in the Custom House, under W. D. Gallagher, Esq., collector of the port.

In 1841 he married a Miss Browne, sister of the late J. Ross Browne.

In 1848 he was again a widower, with two children, a son and daughter.

In 1851 he married a Miss Harrington, his present wife, in Roxbury, Mass. By her has two daughters, both living.

He was one of the founders of the Union Club of Louisville, a large body of true and tried men, which was largely instrumental in preserving the peace and protecting the city.

After the war ceased, the doctor with his nephew, Dr. Frank Peabody (a former student of his), resumed his profession, and continued in full practice until 1880. After this, he, for the most part relinquished practice, though there were many of his old patients who insisted that he should practice for them, and for such he continued to operate as his strength would warrant until within the last year. The remarkable thing about the operations is that they were not inferior to those of his earlier days. Such was his enthusiasm in his profession, that he kept fully abreast with all the modes and all the new improvements of practice, so that his last operations did not exibit results of failing strength. The enthusiasm which he exibited in regard to his profession was a matter of remark and wonder to all who knew him. He was held in very high esteem by the profession. He has ever been regarded as a man of strong integrity and unswerving justice. The fact that he was elected fourteen successive years as the treasurer of the American Dental Association is evidence of the confidence of the profession in his integrity. At the last meeting of this body he was elected to fill the highest position within its gift, which was an additional mark of appreciation.

He was modest and unpretending in his relations with his professional brethren, yet manly in asserting his opinions; practical and steadfast in defending his convictions of the right; honors sought him and were received with appreciation; never, however, did they exercise an undue influence upon him. He was one of nature's noblemen, with purposes true and faithful. As a friend he was true and steadfast. With the false and fictitious he had no sympathy or patience.

He, with his family, has for several years past, spent the summer months at the seashore, by this means seeking to regain his health and strength, as well as minister to the comfort and welfare of his family, and avoiding the debilitating weather of his home.

He inherited a predisposition to asthma, which became more and more manifest as the years rolled by. With this affection he was sorely afflicted each winter. During the past winter it was more severe than ever, and took such a hold upon him that its power could not be broken, and he passed away after months of severe suffering, on the morning of March 4th, 1883.

Dr. Goddard's interest, enthusiasm, energy and industry are exhibited in what has thus far been said in his chosen profession, and in the effort which he put forth in behalf of his country in the time of its greatest peril, and to show that these two causes did not exhaust his energy, reference is here made to the work which he did in another direction, by quoting from a communication of a fellow worker, Mr. George W. Morris. In reference to Dr. Goddard he writes as follows:

Since the Order of Odd Fellows was established in Kentucky, in the year 1833, there has never been a truer, more devoted or more enthusiastic member of it, than Dr. Wm. H. Goddard, of Louisville. He united with it in the spring of 1847, joining Azur Lodge No. 25, nearly thirty-six years ago. From that time to the present he has contributed more of his time, energies and talents to the advancement of the principles and true interests of this Order than any man in this jurisdiction. Besides being a member of his own Lodge, he has held membership in Mount Horeb Encampment No. 1, the Veteran Association, the Degree Staff, the Grand Encampment, and the Grand Lodge of Kentucky.

In all these various and important branches of this benevolent Order he was ever the same active and devoted brother.

He was never satisfied unless he was actively employed in the interest of the cause which he loved so well, and which was so dear to his heart, and to which he, with the most self-sacrificing devotions, dedicated the best and most active years of his life.

He was a genuine Odd Fellow in spirit and in truth. He had studied most thoroughly its principles, precepts, and teaching its adaptability for the promotion of good will among men, and its fitness as a minister to the trials and the adversities which are inseparable from human life. And not only so, but he put them in practice in his daily intercourse with his fellow men. He endeavored to conform to the Golden Rule, by doing unto others as he would that they should do unto him.

Possessed by nature with what might be regarded as rather a rough, blunt exterior, yet with a noble and true heart within, which at once commended him to the esteem, respect, and confidence of his brothers, with whom he was regarded as a Prince among Odd Fellows.

Great as was his influence in the Order at large, it was in his own Lodge, where from first to last it was unbounded. His name in it was ever a tower of strength, and his judgment was almost universally acquiesced in. During a period of more than twenty years he occupied the position of Chairman of the Widows' and Orphans' Committee of the Lodge, his associates on it always turned over to him the care and management of them, they being under his immediate charge and direct supervision. And never in the history of the Lodge was this sacred trust more carefully and efficiently administered, as the long line of widows and orphans, its beneficiaries, will most fully attest.

Indeed, it may with truth be said, that he was their sole guardian; as also of the funds set apart for this special use and benefit. And by his zeal, watchfulness and care, together with the rigid economy he displayed in its management, he for many years past was known and designated as the "Old Guard of Azur Lodge," to whom was confided its most important interest.

In a word he was a man of most positive character, and made a direct impression in every circle in which he moved. His removal from them will be most seriously felt, and his place exceedingly difficult to fill.

He filled the full measure of the declaration: "An Honest Man the Noblest Work of God."

## Figures and Tables

**Figure f1:**